# Polycarbonate Nanofiber Filters with Enhanced Efficiency and Antibacterial Performance

**DOI:** 10.3390/polym17040444

**Published:** 2025-02-08

**Authors:** Miren Blanco, Cristina Monteserín, Estíbaliz Gómez, Estíbaliz Aranzabe, Jose Luis Vilas Vilela, Ana Pérez-Márquez, Jon Maudes, Celina Vaquero, Nieves Murillo, Iñaki Zalakain, Leire Ruiz Rubio

**Affiliations:** 1 Unidad de Química de Superficies y Nanotecnología, Fundación Tekniker, Iñaki Goenaga 5, 20600 Eibar, Spain; cristina.monteserin@tekniker.es (C.M.); estibaliz.gomez@tekniker.es (E.G.); estibaliz.aranzabe@tekniker.es (E.A.); 2 Macromolecular Chemistry Group (LQM), Physical Chemistry Department, Faculty of Science and Technology, University of the Basque Country (UPV/EHU), 48940 Leioa, Spain; joseluis.vilas@ehu.eus (J.L.V.V.); leire.ruiz@ehu.eus (L.R.R.); 3 BCMaterials, Basque Center for Materials, Applications and Nanostructures, UPV/EHU Science Park, 48940 Leioa, Spain; 4 Process and Materials Area, TECNALIA, P Mikeletegi 2, 20009 Donostia-San Sebastian, Spain; ana.perez@tecnalia.com (A.P.-M.); jon.maudes@tecnalia.com (J.M.); celina.vaquero@tecnalia.com (C.V.); 5 Calvera Hydrogen Innovation, EIC—Energy Intelligence Center, Edificio Edith Clarke, Serantes Bidea, 48500 Abanto, Spain; nieves.murillo@calvera.es; 6 Departamento de Ingeniería, Universidad Pública de Navarra (UPNA), 31006 Pamplona, Spain; inaki.zalakain@unavarra.es

**Keywords:** electrospun nanofibers, beaded structure, filtration efficiency, antibacterial coating

## Abstract

The need for clean and safe air quality is a global priority that extends to diverse environments, including households, industrial spaces, and areas requiring respiratory personal protection. In this study, polycarbonate (PC) nanofiber filters coated with a coating containing a silver salt were prepared by the electrospinning process and a subsequent dipping–extraction method. These nanofiber filters presented the enhancement of air filtration efficiency and reinforcement of antibacterial properties. The research includes diverse PC filter structures, assessing beaded and non-beaded structures and varying areal weights. The study evaluated filtration efficiency across NaCl particle sizes (50–400 nm) and pressure drop outcomes. In addition, the antibacterial activity of the coated filters against *E. coli* and other coliforms was investigated by the filtration membrane method. Repetitive testing consistently yields high efficiencies, reaching 100% in thicker filters, and minimal air resistance in beaded filters, presenting an advantage over the current systems. Furthermore, the new properties of the filters will enhance environmental safety, and their time of use will be increased since they prevented the growth of bacteria, and no significant colonies were seen. Considering all these factors, these filters presented promising application in environments with harmful microorganisms, for the development of advanced industrial filtering systems or even hygienic masks.

## 1. Introduction

Environmental pollution is proven to be the leading cause of disease and premature death in the world today [1,2]. The infectious route of a wide range of pathogens is the inhalation of the airborne particles containing the pathogen, which can suspend in the air and retain infectious activity for many hours or days. Moreover, air pollutants from particulate matter can cause respiratory disease especially in people who are sensitive to the suspended dust or have a low pulmonary function [3]. The concern of the society is increasing with the increase in human exposure to air pollution, including pathogens and particles, mainly to nanoparticles, and their presence is increasing as a result of the combustion of carbon-based energy [4]. Therefore, there is a significant demand for filter media capable of efficiently trapping these harmful fine particles as well as other compounds harmful to health.

Various methods, including electric precipitators, wet scrubbers, cyclonic separators, fabric filters, and catalytic filters, have been employed for capturing and eliminating particles and pathogens [5,6,7]. Among these, membrane filtration stands out as the current leading physical method for safeguarding against air pollutants [8]. Nevertheless, the production of filters that exhibit a low-pressure drop, a high-quality factor, and possess antibacterial properties while they are durable and effective remains a significant challenge [9]. Fibrous membranes are a good option for particle filtration due to their cost-effectiveness and energy savings. However, traditional fibers such as glass fibers, melt-blown fibers, and spun fibers, widely used in various air filtration applications, exhibit relatively lower filtration efficiency when dealing with fine particles. This is mainly attributed to the microsized diameter of the fibers and the substantial size of the pores [10].

Nanofibrous membranes fabricated from electrospinning technology have attracted particular interest due to their distinct features including high filtration efficiency, dust-holding capacity, and air permeability [11]. Modern air filter materials, such as those obtained from electrospinning, outperform their traditional counterparts in several aspects [6]. They have large specific surface area, higher porosity, small pore size, and excellent pore connectivity. Thus, nanofiber membranes demonstrate superior filtration performances compared to the traditional filtration materials by measuring the penetration of sodium chloride (NaCl) nanoparticles [12,13]. Beyond these attributes, its substantial scalability potential is noteworthy, offering prospects for higher productivity, greater flexibility, and mechanical robustness [14]. Additionally, these materials exhibit versatility over a wide temperature range, ensuring long life, and they can be used for bacterial and viral removal [6,15]. However, nanofibrous membranes could also present a problem of an excessive pressure drop when filtering fine particles that needs to be solved [10]. There are several potential polymers that could be used for fiber fabrication. However, polycarbonate (PC) stands out among many promising engineering polymers due to its unique mechanical and physical properties. These include chemical resistance, high impact strength, and rigidity, making the nanofibers robust and long-lasting, with toughness and stable performance in high-temperature environments. Moreover, PC nanofibers exhibit high filtration efficiency due to their ability to intercept and capture particles through mechanisms like inertial impaction and diffusion [16,17].

On the other hand, when using these nanofiber membranes in filtering applications related to respiratory protection and indoor air purification, it is very important to display antimicrobial properties. Antimicrobial agent remedy is one of possible solutions to block airborne infections. Silver, over the years, has been used in various antimicrobial applications from water treatment to healthcare and bioengineering [18]. Due to the electrostatic interaction between bacterial cells (−) and silver ions (+), the rupture in the cell wall causes leaking of internal cell content and eventual death of the bacteria [19]. Nanofibers have been produced by adding silver (Ag) directly into the electrospinning polymer solutions using silver in different forms, salt or nanoparticles. However, they showed a decrease in antimicrobial efficiency ascribed to diffusion limitations and/or particle aggregation and subsequently reduced bioavailability [20,21]. The incorporation of the antimicrobial functionality as a coating could be an interesting alternative if the coating does not significantly affect the pressure drop [19].

In this study, highly filtering efficient filters with antibacterial properties have been prepared by using a two-step process. Firstly, nonwoven electrospun nanofiber PC filters were obtained by electrospinning, controlling the parameters of the process to obtain fibers with randomly distributed beads. Thereafter, the bead-containing filters were covered with a silver sol–gel nanocoating via immersion, with the coating being deposited on the individual fibers without significantly affecting their filtration capability. The antibacterial efficiency of the Ag-coated PC filter has been validated according to the ISO 9308-1:2014 standard [22], by analyzing its capability to remove *E. coli* and other coliform bacteria in artificially inoculated water kept in contact with the filter for 24 h. In addition, the filtration efficiency of coated filters has been evaluated.

## 2. Materials and Methods

### 2.1. Materials

PC pellets (Iupilon S-3000) were purchased from Mitsubishi Engineering-Plastics Corp (Düsseldorf, Germany). N,N-dimethylformamide (DMF; 99.8%), tetrahydrofuran (THF; 99.9%), titanium (IV) isopropoxide (TTIP, 7%), acetylacetone (AcacH, 99%), and tetrabutylammonium chloride (TBAC; 99%) were bought from Sigma Aldrich (Steinheim, Germany). Ethanol (EtOH) was obtained from Scharlau and silver nitrate (AgNO_3_, 99%) from Acros Organics (Geel, Belgium). All chemicals were of analytical grade and used without further purification.

### 2.2. PC Electrospun Nanofiber Filter Preparation

PC nanofiber filters were fabricated using a home-made electrospinning unit. The unit consisted of a high voltage (30 kV) power supply (Matsusada Precision Inc. (Kusatsu, Japan), AU-30R1-L), a syringe pump, a grounded collector, a metallic needle connected to the high voltage power supply, and a dehumidifying air system to adjust the humidity.

To facilitate a comparative analysis, two PC filters, denoted as PC-A and PC-B, were fabricated using different solutions: solution A and B.

In the case of PC-A, the objective was to introduce beads throughout the filter volume, enhancing its quality factor. This involved using a 20% weight solution of PC in a 50/50 THF/DMF solvent mixture. The optimized electrospinning conditions for PC-A were an applied voltage of 10 kV, a needle–collector distance of 150 mm, and a flow rate of 0.5 mL/h.

On the other hand, PC-B filters were fabricated with a standard uniform nanofiber structure, avoiding bead formation or thickening, using a 20% weight solution of PC and 1% weight of TBAC salt in a 50/50 THF/DMF solvent mixture. The optimized electrospinning conditions for PC-B were a 15 kV applied voltage, a 150 mm needle–collector distance, and a flow rate of 0.1 mL/h.

Both processes were carried out with a needle of 0.6 mm internal diameter. All tests were performed at room temperature and a humidity between 40 and 50%. Fibers were collected on a polypropylene spunbond nonwoven fabric fixed to the collector to ensure easy detachment of the filter samples. The deposition time were 3 h for the PC-B filters and 3 h for the PC-A filters with nanofiber-bead morphology to evaluate the influence of the filter areal weight on the filtration capacity.

### 2.3. Antibacterial Electrospun Nanofiber Filter Preparation

PC-A filters with beads were coated with an Ag-doped TiO_2_ coating prepared using the sol–gel method, to provide them the antibacterial capacity. Titanium (IV) isopropoxide (TTIP) was used as a precursor and acetylacetone (AcacH) as a stabilizing agent, since metal oxides are highly reactive with water, in a TTIP:AcacH molar ratio of 1:1 (3.6 g TTIP and 1.3 g AcacH), when magnetically stirred for 1 h at room temperature. The solvent of the solution was pure ethanol (EtOH) from Scharlau, and it was used in a TTIP:EtOH molar ratio of 1:200 (79.2 g). AgNO_3_ was used as silver doping agent in a concentration of 0.01 wt.% with respect to the Ti precursor (0.037 g). The silver salt was dissolved in 0.1 N HCl in distilled water (DI) in a TTIP:DI molar ratio of 1:2 (2.51 g DI), and then added to the titania solution drop by drop.

The resultant transparent solution was maintained under magnetic stirring for 1 day. The Ag-TiO_2_ coatings were deposited on the filters by automatic dip coating with a withdrawal speed of 300 mm/min. Then, the coated filters were heat treated at 80 °C for 15 h.

### 2.4. PC Electrospun Nanofiber Membrane Characterization

#### 2.4.1. Microstructural Characterization

The morphology and topography of nanofibrous materials have been observed by scanning electron microscopy (SEM) using an Ultra Gemini-II microscope (Carl Zeiss, Jena, Germany) equipped with Energy-Dispersive X-ray Spectroscopy (EDS), which has been employed for element mapping analysis of nanofibrous surfaces. Samples were analyzed without being coated with gold. The average diameter of the filters was calculated by Image-J software (1.53 t) measuring 100 fiber diameters in the SEM images. The areal weight density of the electrospun nanofiber filters was determined by weighing a 10 × 10 cm area in a balance with an accuracy of 0.0001 g.

#### 2.4.2. Particle Filtration Performance of Electrospun PC and PC-Ag Filters

The experimental method used in the filtration tests to evaluate the performance of the filters was based on standard UNE-EN 1822-3 [23]. The experimental system used in the tests includes three main parts: the aerosol generation unit, the installation supporting the filter, and the particle measuring equipment before and after the filter.

A polydispersed aerosol was generated from a 0.1% NaCl solution with a collision-type atomizer. To eliminate moisture, the produced aerosol was passed through a Nafion membrane dryer. Subsequently, the dry aerosol underwent neutralization by a bipolar ionizer, acquiring a Boltzmann distribution, before entering the differential mobility analyzer (DMA). In the DMA, particles were sorted based on their aerodynamic size, allowing the generation of a monodisperse aerosol.

At the outlet of the differential mobility analyzer (DMA), the monodisperse aerosol flow was split into two streams. One stream was directed to a condensation particle counter (CPC-1) to measure the particle concentration before entering the filter. The second stream was routed to the filter holder, which housed the test filter. The filtered aerosol was analyzed downstream using CPC-2 to determine the concentration of particles that passed through the filter. Simultaneously, a Magnehelic gauge continuously monitored the pressure drop across the filter holder, ensuring the accurate tracking of resistance. Throughout the process, the flow rates were carefully controlled to maintain consistent testing conditions.

The aerosol generator and the pre-filtration concentration measuring apparatus were included in the aerosol generator and monitor, and AGM (MSP Model 1500, MSP, Inc., Shoreview, MN, USA) equipment and the post-filtration particle measuring apparatus were included in the humidified tandem differential mobility analyzer, HTDMA, (MSP model 1040XP, MSP, Inc., Shoreview, MN, USA) equipment.

The tested filters had a diameter of 47 mm. They were tested in a 47 mm Millipore filter holder, model XX4404700 (Millipore Corporation, Bedford, MA, USA), and its filtration area was 13.8 cm^2^.

The tests were performed with a monodisperse aerosol of 50/100/200/300/400 nm particle size, and for each sample, the test was repeated three times. Considering the filtration area, the speed through the filtration medium was 0.5 cm/s. The mean particle penetration (Pe) and mean filtration efficiency (E) through the filters were calculated by means of Equations (1) and (2).

Pe = C_out_/C_in_ (1)

E = 1 − Pe(2)

Wherein, C_out_ is the mean concentration measured after the filter with CPC-2, and C_in_ is the mean concentration measured before the filter with CPC-1.

The quality factor (Q_f_) or figure of merit has been further calculated according to Equation (3), where Pe is the mean particle penetration and ∆p is the pressure drop.

Q_f_ = ln(1/Pe)/∆p = − ln(Pe)/∆p (3)

The results were presented in % of filtration efficacy for each particle size between 100 and 400 nm. For each sample, the mean value of efficacy as well as the minimum and maximum values of three tests performed with each of the samples have been depicted.

#### 2.4.3. Pressure Drop

The pressure drop across the thickness of nanofiber filters was measured by a digital manometer (Testo 510, Testo SE & Co. KGaA, Baden-Württemberg, Germany). Pressure drop experiments were carried out to study the behavior of the nanofiber filters compared with the commercial filter at varied air face velocities in the range of 0.1 and 25 cm/s, based on the filtration efficiency test standards used by the American Society for Testing and Materials [24,25].

#### 2.4.4. Antibacterial Tests of PC Modified Electrospun Nanofiber Filters

The antibacterial capacity of the nanofiber filters was characterized by Microinstant^®^ CCA Chromogenic Coliform Agar (Scharlab, Barcelona, Spain) kit that complied with the formula described in the ISO 9308-1:2014 standard [22]. This is a selective and differential medium for detecting *E. coli* and total coliforms in water samples by the filtration membrane method [26]. Initially, deionized water was artificially inoculated with fecal water to introduce a concentration of *E. coli* and other coliform bacteria suitable for measurement. An area of 4 × 4 cm of the polycarbonate nanofiber filters (PC-A) with and without the silver coating were placed in contact with 100 mL of the inoculated H_2_O for 24 h. During this contact time, the solutions were kept in darkness. After this time, the inoculated water was filtered through a membrane with a pore diameter of 0.45 µm, according to the established method. The membrane was deposited face up on a plate containing the ACC medium, endeavoring not to form bubbles or creases. The plate with the membrane was incubated for 18–24 h at 36 ± 2 °C. If at 18 h red or colorless colonies appear, incubation was prolonged up to 24 h to include the possible late reactions of β-galactosidase or of β-glucuronidase. After the incubation period, colonies having a color ranging from salmon pink to red were counted as coliform bacteria other than *E. coli*, and those having colors ranging from dark blue to violet were counted as *E. coli*. The total coliform bacteria count corresponds to the sum of colonies ranging in color from salmon pink to red and the colonies ranging in dark blue to violet. During the evaluation of the membranes, a photograph has been taken after 24 and 48 h of incubation.

## 3. Results and Discussion

### 3.1. PC Nanofiber Filter Microstructure Characterization

**Figure 1:**
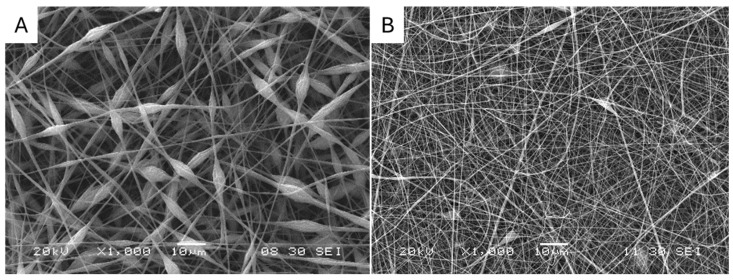
SEM images of fabricated electrospun filters showcasing their microstructure and fiber morphology: (**A**) PC-A and (**B**) PC-B.

PC nanofiber filters were produced using a custom electrospinning unit. Two formulations were used resulting in two different morphologies, PC-A and PC-B; see Figure 1. PC-A incorporated beads throughout the filter, enhancing its quality factor, while PC-B featured a uniform nanofiber structure without beads. For the fiber formulations, PC-A used a 20% weight PC solution in a 50/50 THF/DMF solvent mix, whereas PC-B used a 20% weight PC solution with 1% weight of TBAC salt in the same solvent mix. Scanning Electron Microscope (SEM) results provided insight into the morphological characteristics of the fibers. The PC nanofiber and bead diameters and bead density for the PC-A were calculated with the Image-J image analysis program. The PC-A filter showed an average fiber diameter of 445 nm ± 98 nm and an average bead diameter of 3.74 ± 0.65 µm, having a density of around 1 million beads per cm^2^. The TBAC salt added in the PC-B filter solution generates an increase in the solution conductivity, resulting in a beadless morphology and a reduction in the fiber diameter, having an average diameter of 250 ± 80 nm. An increase in solution conductivity might have enabled the solution to carry more charge leading to a stronger electrostatic force corresponding to the stretching of the fiber. This would explain having a morphology without beads and finer fibers for the PC-B filter.

Several authors have described interesting results of membranes with the beads-on-the-string (BOTS) fibrous morphology [27]. BOTS fibers have been applied in various fields, including drug delivery [28], superhydrophobic materials [29], and distillation [30]. In addition, BOTS fibers have also shown promising results when used in air filtration applications. An example of the successful use of this BOTS, Li et al. fabricated graphene oxide/polyacrylonitrile (GOPAN) nanofibrous membranes with an olive-like beads-on-a-string structure via electrospinning with excellent PM2.5 removal efficiency and low pressure drop for air purification applications [31]. Similarly, the studies conducted by Huang et al. highlighted that the bead-like configuration is more effective for air filtration when using polyacrylonitrile as a substrate [32]. Considering the potential of BOTS fibers, PC-A fibers were selected for the rest of the study.

**Figure 2:**
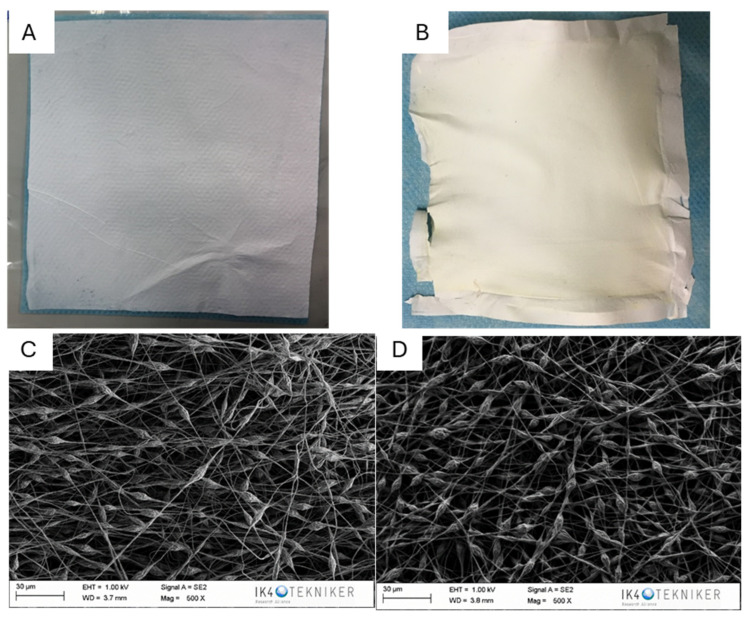
Photographs and SEM images of fabricated PC-A electrospun filters: (**A**,**C**) uncoated and (**B**,**D**) coated with the silver-containing formulation.

Selected PC-A filters with beads were coated with an Ag-doped TiO_2_ layer using a sol–gel method to provide antibacterial properties. The coating was applied via dip coating and heat-treated to ensure adhesion. The coating was characterized by SEM and EDS. Figure 2 shows photographs and SEM images of the untreated and coated PC-A filters.

**Figure 3:**
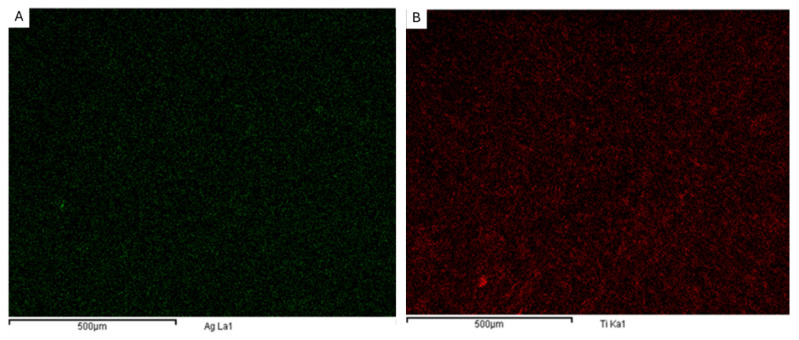
EDS mapping of coated electrospun fibers: (**A**) silver in green and (**B**) titania in red.

As it can be observed in the images, the coated substrate changed slightly to the naked eye. The coating increased the density of the filter at a micrometric size, with the increase in the areal weight being remarkable from 1.9 to 4.3 g/m^2^. In addition, the homogeneity of the coating was assessed by the EDS mapping of the elemental silver and titania corresponding to the Ag-doped TiO_2_ coating. The result can be observed in Figure 3 and Table 1.

The EDS analysis confirms the presence of the main elements: carbon and oxygen from polycarbonate and titanium (Ti) and silver (Ag) from the TiO_2_ coating. The results corroborate that TiO_2_ is a significant component of the coating, whereas the presence of silver in trace amounts is consistent with the doping of TiO_2_. On the other hand, SEM/EDS for Ag and Ti confirm the homogeneity of the coating in the surface of the filter.

### 3.2. Evaluation of the Antimicrobial Activity of the PC-A Nanofiber Filters

The antibacterial capacity of the filters with respect to *E. coli* and other coliform bacteria present in water was characterized by means of the Microinstant^®^ CCA Chromogenic Coliform Agar kit. For the preparation of the inoculated water, a ratio of fecal water/deionized water of 1:1000 was employed. After 24 h of contact between the PC-A nanofiber filters and the artificially inoculated water, the water volume was filtered through the test membranes, and the membranes were incubated at 36 ± 2 °C. Table 2 shows the final appearance of the membranes after 24 and 48 h of incubation. 

After 24 or 48 h of incubation at 36 °C, the membranes that were in contact with the artificially inoculated water or the artificially inoculated water and the PC-A nanofiber filter presented dark blue, purple, and pink colored colonies of bacteria, indicating the simultaneous action of the enzymes present in *E. coli* and other coliform bacteria. The membranes in contact with the artificially inoculated water and the Ag-coated PC-A nanofiber filter did not present any bacteria, even after 48 h of incubation. This fact confirms the antibacterial action of the filter, which is capable of preventing the growth of bacteria without the action of UV radiation.

### 3.3. Evaluation of the Filtration of the PC-A Nanofiber Filters

**Figure 4:**
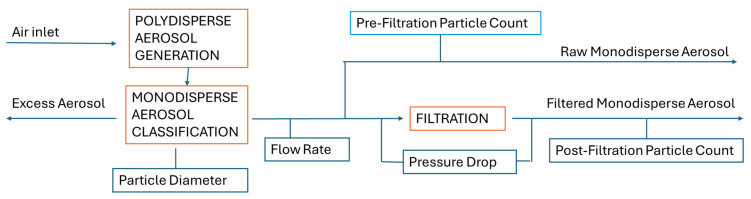
The scheme of the monodisperse aerosol flow generation and the monitoring.

Once the antibacterial capacity of the PC-A filters was validated, particle filtration tests to measure the efficiency and pressure drop of these filters, both with and without the coating, were carried out. In addition, the particle filtration tests were performed in PC-B for comparison purposes. The tests were performed using a test bench, as shown in Figure 4, which requires the generation of a monodisperse aerosol flow. During the tests, the particle diameter was measured both before and after filtration, the flow rate of all involved streams was recorded, and the pressure drop resulting from the passage through the filter was also determined.

**Figure 5:**
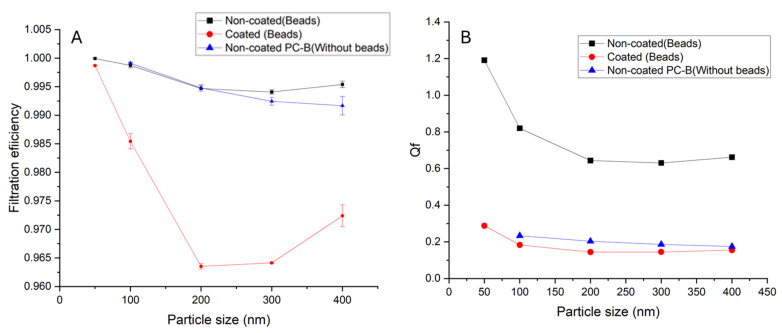
(**A**) Filtration efficiency and (**B**) quality factor of the non-coated and coated filters.

In a first analysis, the filtration efficiencies of the filters were evaluated using NaCl test particles of 50–400 nm in diameter; see results in Figure 5. The use of NaCl (sodium chloride) particles in filtration tests is widely adopted due to both technical and regulatory reasons. NaCl particles are chemically stable, easily atomized into uniform sizes, and widely used in international standards such as UNE-EN 1822-3 [23], which is the standard followed in the paper. They effectively simulate respirable particles (<0.3 µm) found in the air, enabling precise evaluation around the most penetrating particle size (MPPS). In addition, the optical and electrical properties of the NaCl particles could simplify their detection using photometers or particle counters, ensuring reliable results. Additionally, NaCl is cost-effective, safe, and widely available, making it ideal for controlled experiments, despite its simpler composition compared to complex, real-world aerosols.

As evident from Figure 5A, the filtration efficiency of PC-B (without bead) is lower than for the PC-A (with beads), mainly for bigger particles. Moreover, while the uncoated filters demonstrate greater effectiveness in filtering particles, those coated with Ag-doped TiO_2_-coated filters are also effective, achieving a minimum filtration efficiency of 0.964, a notably high value [33,34] in comparison with other reported values for polycarbonate fibers. Moreover, a similar trend is observed in both cases: the filters exhibit the highest efficiency for filtering smaller particles, around 50 nm, and their lowest efficiency for particles measuring 200 to 300 nm. The traditional theories in the filtration of gas stream for solid particles removal by fibers are explained by direct interception, Brownian diffusion, inertial impaction, gravity settling, or electrostatic deposition, which depend on particle sizes, gas velocities, fiber diameters, etc. [26]. Electrospun filters are less effective for large particles because they have greater mass and inertia, reducing the likelihood of being captured by the nanofibers. Moreover, the pores between the electrospun fibers are often large enough to allow larger particles to pass through. On the other hand, electrospun filters are more suitable for capturing ultrafine particles because their nanofiber structure offers a high surface-to-volume ratio and pore size optimized for trapping small particles. They utilize mechanisms like diffusion (Brownian motion) and electrostatic attraction, which are particularly effective for ultrafine particles that move randomly or are influenced by electrical charges.

The analysis of the quality factor yield results was similar to those observed for filtration efficiency; see Figure 5B. The Q_f_ was higher for PC-A with beads, showing the clear influence in performance of the presence of the beads. On the other hand, the Q_f_ of the uncoated filters shows significantly higher values than the coated filter. In both cases, the Qf for 50 nm particles is the highest value. However, in the case of the uncoated filter, the quality factor decreases rapidly until stabilizing at 0.5 for particles of 200 nm and larger. On the other hand, for the coated filter, while a decrease is also observed, it is significantly less pronounced, remaining nearly stable. This indicates that the coating has minimized the dependence of particle–filter interactions on particle size. Overall, the difference between the two filters in terms of filtration efficiency and quality factor is more pronounced for particles smaller than 200 nm, where the uncoated filter is more efficient. However, this superiority is less evident for particles larger than 200 nm. Thus, the coating appears to have slightly reduced its filtration capacity, with minimum values of 0.995 and 0.965 for uncoated and coated filters, respectively, although it exhibits excellent antibacterial properties.

The decreased dependence of particle–filter interactions on size after coating could be related to changes in the physical and chemical properties of the filter. Usually, large particles filtration relies on size-dependent mechanisms such as inertial impaction and direct interception, which require open pores and rough fiber surfaces. In this case, the coating smoothens the fibers, reduces pore size, and partially obstructs airflow, significantly impairing these mechanisms by reducing the large particle filtration capacity. On the other hand, small particle filtration, commonly driven by Brownian diffusion and electrostatic attraction, is less affected by pore structure or fiber texture. As long as the coating maintains the charge of the fibers and surface chemistry, small particle filtration efficiency could remain more stable. The Qf was calculated with the pressure drop measured at 0.5 cm/s face velocity, with ∆p being 8.13 Pa, 23 Pa, and 78.5 Pa for non-coated, coated, and without beads filters, respectively.

**Figure 6:**
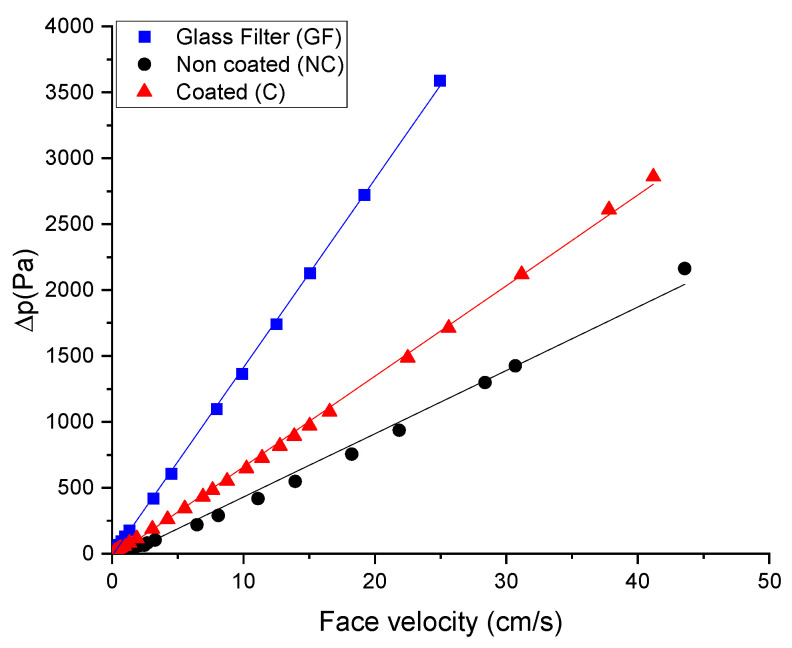
Pressure drop versus face velocity plot for non-coated and coated filters.

The developed membranes exhibit a linear relationship, similar to the commercial glass filter. This indicates that the pressure drop increases proportionally with face velocity for all three materials [35]. This filter shows a medium slope, that is, it has a pressure drop lower than the glass fiber but higher than the uncoated filter. Its behavior may be due to an intermediate structure in terms of porosity and thickness of the filter. The coating could reduce the effective size of the pores or increase the resistance to flow as shown in Figure 6. On the other hand, the uncoated sample has the lowest slope, which means that it generates the lowest resistance to flow and the lowest pressure drop for the same face speed. This seems to corroborate that the pores are larger than the coated fibers, and that the structure is less dense, which has been already observed in the SEM images.

## 4. Conclusions

Antibacterial high-efficiency filters have been successfully prepared by using a two-step process. Firstly, nonwoven electrospun PC nanofiber filters were obtained by electrospinning resulting in two morphologies: PC-A and PC-B. PC-A featured bead-like structures within the nanofibers, enhancing its quality factor, with average fiber and bead diameters of 445 nm ± 98 nm and 3.74 µm ± 0.65 µm, respectively. In contrast, PC-B displayed a beadless morphology and finer fibers with an average diameter of 250 nm ± 80 nm. Thereafter, PC-A filters were chosen for further analysis due to their superior performance in filtration tests, as shown in previous studies. PC-A was covered with a silver-containing nanocoating via immersion.

The antibacterial efficiency of the Ag-coated PC-A filter has been validated according to the ISO 9308-1:2014 standard [22], by analyzing its capability to remove *E. coli* and other coliform bacteria in an artificially inoculated water kept in contact with the filter. After 24 and 48 h of incubation, the Ag-doped TiO_2_-coated filters showed no bacterial growth, confirming their strong antibacterial properties, even without UV radiation. This outcome demonstrates that the coating effectively prevents bacterial colonization, making these filters suitable for applications requiring sterilization and pathogen control.

Filtration tests using NaCl particles (50–400 nm) revealed that nonwoven electrospun nanofiber PC-B (without bead) has lower efficiency than PC-A (with beads), mainly for bigger particles. PC-A filters, both coated and uncoated filters, achieved high filtration efficiencies (>96%). PC-A filters presented high filtration capacity, with minimum values of 0.995 and 0.965 for uncoated and coated filters, and pressure drops increased proportionally with face velocity and had values of 8.13 Pa and 23 Pa for non-coated and coated filters, respectively. The uncoated filter is significantly more effective in terms of balancing filtration efficiency and pressure drop. The coating increased airflow resistance, resulting in higher pressure drops compared to uncoated filters. Nevertheless, it is important to emphasize that the filtration efficiency of the membrane coated with Ag-doped TiO_2_ remains high, exceeding 0.965, and it possesses excellent antibacterial properties, making them advantageous for multifunctional filtration applications.

In summary, the Ag-coated PC electrospun filters with bead-like structures within the nanofibers have been demonstrated to be reliable for the removal of *E. coli* and other coliforms, maintaining a high-efficiency filtration capacity, and these filters are suitable for the development of advanced industrial filtering systems or even hygienic masks, thus contributing in this way to improving air quality and people’s health and protecting them from pollution and potentially harmful aerosols.

## Figures and Tables

**Table 1:** The analysis of elemental composition of the coated filter by SEM/EDS.

Element	Weight%	Atomic%
C K	64.00	73.99
O K	27.13	23.54
Si K	0.08	0.04
Cl K	0.14	0.05
Ca K	0.10	0.03
Ti K	7.63	2.21
Cu K	0.12	0.03
Ag L	0.80	0.10
Total	100.00	

**Table 2:** Photographs of the filters after 24 h and 48 h of incubation at 36 ± 2 °C.

Incubation Time (h)	Reference Inoculated Water 1:1000	PC-A Electrospun Nanofiber Filter	Ag-coated PC-A Electrospun Nanofiber Filter
24	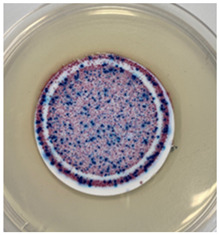	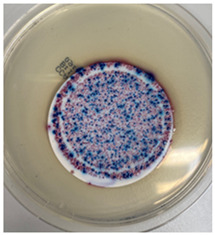	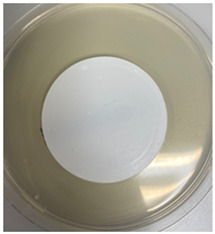
48	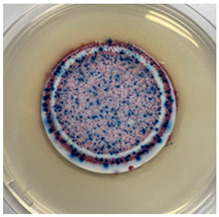	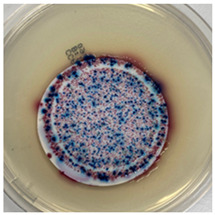	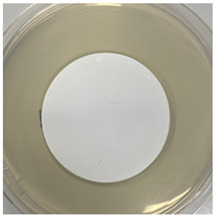

## Data Availability

The original contributions presented in this study are included in the article. Further inquiries can be directed to the corresponding author.

## References

[1] Franklin B.A., Brook R., Arden Pope C. (2015). Air Pollution and Cardiovascular Disease. Curr. Probl. Cardiol..

[2] Takizawa H. (2011). Impact of Air Pollution on Allergic Diseases. Korean J. Intern. Med..

[3] Kyung S.Y., Jeong S.H. (2020). Particulate-Matter Related Respiratory Diseases. Tuberc. Respir. Dis..

[4] Perera F., Ashrafi A., Kinney P., Mills D. (2019). Towards a Fuller Assessment of Benefits to Children’s Health of Reducing Air Pollution and Mitigating Climate Change Due to Fossil Fuel Combustion. Environ. Res..

[5] Bolashikov Z.D., Melikov A.K. (2009). Methods for Air Cleaning and Protection of Building Occupants from Airborne Pathogens. Build. Environ..

[6] Bortolassi A.C.C., Nagarajan S., de Araújo Lima B., Guerra V.G., Aguiar M.L., Huon V., Soussan L., Cornu D., Miele P., Bechelany M. (2019). Efficient Nanoparticles Removal and Bactericidal Action of Electrospun Nanofibers Membranes for Air Filtration. Mater. Sci. Eng. C Mater. Biol. Appl..

[7] Ren H., Koshy P., Chen W.F., Qi S., Sorrell C.C. (2017). Photocatalytic Materials and Technologies for Air Purification. J. Hazard. Mater..

[8] Komaladewi A.A.I.A.S., Aryanti P.T.P., Subagia I.D.G.A., Wenten I.G. (2019). Membrane Technology in Air Pollution Control: Prospect and Challenge. J. Phys. Conf. Ser..

[9] Zhu M., Han J., Wang F., Shao W., Xiong R., Zhang Q., Pan H., Yang Y., Samal S.K., Zhang F. (2017). Electrospun Nanofibers Membranes for Effective Air Filtration. Macromol. Mater. Eng..

[10] Wang Z., Pan Z., Wang J., Zhao R. (2016). A Novel Hierarchical Structured Poly(Lactic Acid)/Titania Fibrous Membrane with Excellent Antibacterial Activity and Air Filtration Performance. J. Nanomater..

[11] Zhou Y., Liu Y., Zhang M., Feng Z., Yu D.-G., Wang K., Arias J.L., Scherf U., El-Hammadi M.M., Zhou Y. (2022). Electrospun Nanofiber Membranes for Air Filtration: A Review. Nanomaterials.

[12] Matulevicius J., Kliucininkas L., Prasauskas T., Buivydiene D., Martuzevicius D. (2016). The Comparative Study of Aerosol Filtration by Electrospun Polyamide, Polyvinyl Acetate, Polyacrylonitrile and Cellulose Acetate Nanofiber Media. J. Aerosol Sci..

[13] Al-Attabi R., Dumée L.F., Schütz J.A., Morsi Y. (2018). Pore Engineering towards Highly Efficient Electrospun Nanofibrous Membranes for Aerosol Particle Removal. Sci. Total Environ..

[14] Buivydiene D., Krugly E., Ciuzas D., Tichonovas M., Kliucininkas L., Martuzevicius D. (2019). Formation and Characterisation of Air Filter Material Printed by Melt Electrospinning. J. Aerosol Sci..

[15] Fahimirad S., Fahimirad Z., Sillanpää M. (2021). Efficient Removal of Water Bacteria and Viruses Using Electrospun Nanofibers. Sci. Total Environ..

[16] Baby T., Jose T.E., Aravindkumar C.T., Thomas J.R. (2021). A Facile Approach for the Preparation of Polycarbonate Nanofiber Mat with Filtration Capability. Polym. Bull..

[17] Li Q., Xu Y., Wei H., Wang X. (2016). An Electrospun Polycarbonate Nanofibrous Membrane for High Efficiency Particulate Matter Filtration. RSC Adv..

[18] Vanangamudi A., Hamzah S., Singh G. (2015). Synthesis of Hybrid Hydrophobic Composite Air Filtration Membranes for Antibacterial Activity and Chemical Detoxification with High Particulate Filtration Efficiency (PFE). Chem. Eng. J..

[19] Park K., Kang S., Park J.-w., Hwang J. (2021). Fabrication of Silver Nanowire Coated Fibrous Air Filter Medium via a Two-Step Process of Electrospinning and Electrospray for Anti-Bioaerosol Treatment. J. Hazard. Mater..

[20] Shi Q., Vitchuli N., Nowak J., Caldwell J.M., Breidt F., Bourham M., Zhang X., McCord M. (2011). Durable Antibacterial Ag/Polyacrylonitrile (Ag/PAN) Hybrid Nanofibers Prepared by Atmospheric Plasma Treatment and Electrospinning. Eur. Polym. J..

[21] Selvam A.K., Nallathambi G. (2015). Polyacrylonitrile/Silver Nanoparticle Electrospun Nanocomposite Matrix for Bacterial Filtration. Fibers Polym..

[22] (2014). Water Quality—Enumeration of Escherichia coli and Coliform Bacteria, Part 1: Membrane Filtration Method for Waters with Low Bacterial Background Flora.

[23] (2010). High Efficiency air Filters (EPA, HEPA and ULPA)—Part 3: Testing Flat Sheet Filter Media.

[24] (2017). Test Method for Determining the Initial Efficiency of Materials Used in Medical Face Masks to Penetration by Particulates Using Latex Spheres.

[25] O’Kelly E., Pirog S., Ward J., Clarkson P.J. (2020). Ability of Fabric Face Mask Materials to Filter Ultrafine Particles at Coughing Velocity. BMJ Open.

[26] Blanco M., Monteserín C., Angulo A., Pérez-Márquez A., Maudes J., Murillo N., Aranzabe E., Ruiz-Rubio L., Vilas J.L. (2019). TiO_2_-Doped Electrospun Nanofibrous Membrane for Photocatalytic Water Treatment. Polymers.

[27] Yang Y., Chen W., Wang M., Shen J., Tang Z., Qin Y., Yu D.G. (2023). Engineered Shellac Beads-on-the-String Fibers Using Triaxial Electrospinning for Improved Colon-Targeted Drug Delivery. Polymers.

[28] Li T., Liu L., Wang L., Ding X. (2019). Solid Drug Particles Encapsulated Bead-on-String Nanofibers: The Control of Bead Number and Its Corresponding Release Profile. J. Biomater. Sci. Polym. Ed..

[29] Rasouli M., Pirsalami S., Zebarjad S.M. (2020). Study on the Formation and Structural Evolution of Bead-on-String in Electrospun Polysulfone Mats. Polym. Int..

[30] Zhao Q., Yu S., Zhu J., Gong G., Hu Y. (2024). Beads-on-String Structural Nanofiber Membrane with Ultrahigh Flux for Membrane Distillation. Sep. Purif. Technol..

[31] Li J., Zhang D., Yang T., Yang S., Yang X., Zhu H. (2018). Nanofibrous Membrane of Graphene Oxide-in-Polyacrylonitrile Composite with Low Filtration Resistance for the Effective Capture of PM2.5. J. Memb. Sci..

[32] Huang J.J., Tian Y., Wang R., Tian M., Liao Y. (2020). Fabrication of Bead-on-String Polyacrylonitrile Nanofibrous Air Filters with Superior Filtration Efficiency and Ultralow Pressure Drop. Sep. Purif. Technol..

[33] Soo J.C., Monaghan K., Lee T., Kashon M., Harper M. (2016). Air Sampling Filtration Media: Collection Efficiency for Respirable Size-Selective Sampling. Aerosol Sci. Technol..

[34] Cho B.M., Nam Y.S., Cheon J.Y., Park W.H. (2015). Residual Charge and Filtration Efficiency of Polycarbonate Fibrous Membranes Prepared by Electrospinning. J. Appl. Polym. Sci..

[35] Huang H.L., Yang S. (2006). Filtration Characteristics of Polysulfone Membrane Filters. J. Aerosol Sci..

